# Phase I dose-finding study of monotherapy with atezolizumab, an engineered immunoglobulin monoclonal antibody targeting PD-L1, in Japanese patients with advanced solid tumors

**DOI:** 10.1007/s10637-016-0371-6

**Published:** 2016-07-01

**Authors:** Hidenori Mizugaki, Noboru Yamamoto, Haruyasu Murakami, Hirotsugu Kenmotsu, Yutaka Fujiwara, Yoshimasa Ishida, Tomohisa Kawakami, Toshiaki Takahashi

**Affiliations:** 1 Department of Experimental Therapeutics, Exploratory Oncology Research & Clinical Trial Center, National Cancer Center Hospital, 5-1-1 Tsukiji, Chuo-ku, Tokyo, 104-0045 Japan; 2 Division of Thoracic Oncology, Shizuoka Cancer Center, 1007 Shimonagakubo, Nagaizumi-cho, Sunto-gun, Shizuoka, 411-8777 Japan; 3 Chugai Pharmaceutical Co., Ltd, 2-1-1 Nihonbashi-Muromachi, Chuo-ku, Tokyo, 103-8324 Japan

**Keywords:** Atezolizumab, Pharmacokinetics, Phase I, Solid tumors, Safety

## Abstract

*Background* Atezolizumab is an engineered immunoglobulin monoclonal antibody that targets the programmed death-1/programmed death-ligand 1 pathway. *Methods* In this phase I dose-finding study, we assessed the safety, feasibility, pharmacokinetics (PK), and exploratory anti-tumor activity of atezolizumab monotherapy up to 20 mg/kg in Japanese patients with advanced solid tumors who had failed standard therapy or for whom there is no standard therapy. *Results* Six patients were enrolled and received intravenous atezolizumab every 3 weeks (q3w) at doses of 10 or 20 mg/kg. Tumor types were non-small cell lung cancer (*n* = 3), melanoma (*n* = 1), pancreatic cancer (*n* = 1), and thymic cancer (*n* = 1). No dose-limiting toxicities were observed. All adverse events (AEs) were grade 1 or 2 in severity. No discontinuations or deaths due to AEs were observed. As of the data cutoff, no partial responses were observed; however, stable disease was observed in all six patients. The maximum mean serum atezolizumab concentration was 220 μg/mL (SD ± 21.9), with 10-mg/kg dosing and 536 μg/mL (SD ± 49.4) with 20-mg/kg dosing. Three patients were still on treatment, and three of the six had achieved a progression-free survival of >12 months. *Conclusions* Atezolizumab was well tolerated in Japanese patients at doses up to 20 mg/kg q3w. The safety profile and Cycle 1 serum atezolizumab concentrations were similar to those previously observed in non-Japanese patients. These data support the participation of Japanese patients in ongoing pivotal global studies of atezolizumab.

## Introduction

Programmed death-ligand 1 (PD-L1) is an immune-checkpoint protein within the cancer-immunity cycle that is expressed on the surface of tumor cells (TC) and tumor-infiltrating immune cells (IC) to downregulate T-cell function [1]. PD-L1 binds to the programmed death-1 (PD-1) and B7.1 (CD80) proteins, and this binding can inhibit the killing of tumor cells by the immune system and decrease T-cell activation, migration, and proliferation [1, 2]. Expression of PD-L1 is prevalent among many tumors, including lung cancer, ovarian cancer, melanoma, brain tumors, malignant lymphoma, multiple myeloma, and colon cancer, and its overexpression is associated with poor prognosis in a number of cancers, including renal cancer, melanoma, lung cancer, ovarian cancer, and colon cancer [3–8].

Atezolizumab (TECENTRIQ™, MPDL3280A, F. Hoffmann-La Roche Ltd., Basel, Switzerland/Genentech, Inc., South San Francisco, CA) is an engineered immunoglobulin monoclonal anti-PD-L1 antibody. Inhibiting PD-L1 with atezolizumab can restore the anti-tumor activity of T cells and enhance T-cell priming [9–11]. Because atezolizumab does not inhibit PD-L2/PD-1 interactions, immune homeostasis is presumably also maintained [11–15]. A previous phase Ia study has shown that atezolizumab pharmacokinetics (PK) are consistent with those of typical immunoglobulins, with a mean terminal serum half-life of approximately 3 weeks [9]. Dose-limiting toxicities (DLTs) were not reported, neither was a maximum tolerated dose, indicating that atezolizumab was well tolerated up to 20 mg/kg every 3 weeks [9]. Atezolizumab’s clinical development began outside of Japan in May 2011 for a number of cancers, including advanced or recurrent non-small cell lung cancer and advanced or recurrent renal cell carcinoma. Because atezolizumab may also benefit Japanese patients, the objective of this study was to evaluate the safety, feasibility, PK, and exploratory anti-tumor activity of atezolizumab monotherapy up to 20 mg/kg in Japanese patients with advanced solid tumors.

## Patients and methods

### Patient eligibility

Key inclusion criteria were as follows: Eastern Cooperative Oncology Group Performance Status (ECOG PS) of 0 or 1, age ≥ 20 years, solid tumor, life expectancy ≥12 weeks, advanced or recurrent cancer that was refractory to the standard of care or for which no standard of care exists, measurable disease, ability to provide a minimum of five unstained tumor section slides prior to treatment (stored samples or samples collected after enrollment), adequate bone marrow (white blood cell count >2500/μL and <15,000/μL, absolute neutrophil count ≥1500/μL, lymphocyte count ≥500/μL, platelet count ≥10.0 × 10^4^/μL), hemoglobin ≥9.0 g/dL, hepatic function (total bilirubin ≤1.5 × the upper limit of normal [ULN], aspartate aminotransferase and alanine aminotransferase ≤3.0 × ULN, alkaline phosphatase [ALP] ≤2.5 × ULN), renal function (serum creatinine ≤1.5 × ULN), and coagulation (prothrombin time international normalized ratio and activated partial thromboplastin time ≤1.5 × ULN). The exclusion criteria were as follows: history of anti-CTLA-4, PD-1, or PD-L1 antibody therapy, autoimmune disease, interstitial pneumonia or pulmonary fibrosis, serious pre-existing medical condition (severe heart disease, uncontrolled diabetes, and active infection), hepatitis B or C virus or human immunodeficiency virus infection, pericardial or pleural effusion requiring drainage, and primary central nervous system tumors or symptomatic central nervous system metastases.

### Study design and treatment

This was an open-label, multicenter, dose-escalation phase I study (JO28944; JapicCTI-132208) conducted with two cohorts (Cohort 1: atezolizumab 10 mg/kg every 3 weeks intravenously; Cohort 2: 20 mg/kg every 3 weeks intravenously). Based on the results of the previously reported phase Ia US study, the starting dose of atezolizumab was determined to be 10 mg/kg [9]. Escalation to the higher-dose second cohort was based on the occurrence of DLTs (see definitions below) during the DLT evaluation period (21-day period from Day 1 of Cycle 1), as follows (traditional 3 + 3 design): three patients were entered at the initial dose level. If a DLT was observed in one-third of the patients at this dose level, an additional three patients were entered at the same dose level. The dose level at which at least two patients experienced DLTs was defined as an unacceptable dose of atezolizumab in Japanese patients.

The primary objectives were identification of the AE and DLT profiles and feasibility up to 20 mg/kg of atezolizumab. The definition of DLT (i.e., unacceptable toxicity) was as follows: grade 4 neutropenia persisting ≥7 days or requiring treatment with granulocyte colony-stimulating factor, febrile neutropenia, grade 4 thrombocytopenia or grade 3 decreased platelet count requiring platelet transfusion, grade 4 anemia or grade 3 anemia requiring a red blood cell transfusion, and grade ≥ 3 nonhematologic toxicity, excluding controllable grade 3 nausea, vomiting, or diarrhea that recovered to grade ≤ 1 as a result of treatment prior to infusion in the next cycle. Secondary objectives reported here are PK and anti-tumor activity. PD-L1 expression is also shown (exploratory objective).

### Assessments

AE severity was graded according to the National Cancer Institute’s Common Terminology Criteria for Adverse Events v4.03. All AEs were assessed until 30 days after the final infusion of atezolizumab. However, AEs were investigated until the day before starting alternative treatment if the patient was starting alternative treatment within 30 days after the final infusion of atezolizumab, or until the day of the final clinic visit if the patient was unable to visit the clinic (e.g., due to hospital transfer or patient circumstances).

Tumor lesions were measured and evaluated in accordance with Response Evaluation Criteria in Solid Tumors (RECIST) version 1.1 [16]. Tumor lesions were assessed within 28 days before enrollment, during the treatment period (Day 22 of even-numbered cycles [−7 to +2 days; evaluation was to be completed before infusion in the next cycle. If imaging showed disease progression and the patient finished the study, examination of lesions at completion of the study was not mandatory]), and at the last observation.

### Pharmacokinetics

Serum samples for PK analyses were collected at Cycle 1, Days 1 (before infusion, 30 ± 30 min after infusion), 2 (24 ± 6 h after infusion), 4 (72 ± 12 h after infusion), 8 (± 1 day), 15 (± 1 day), and 22 (± 1 day); Cycles 2, 3, and 4, Days 1 (30 ± 30 min after infusion) and 22 (± 1 day); Cycles 5 and 7, Day 1 (30 ± 30 min after infusion); and Cycle 6 onward, Day 22 of even-numbered cycles (± 1 day). The blood samples were centrifuged at 1500 to 2000×g for 15 min at 4 °C. The serum samples were then stored at −70 °C or less. The concentrations of atezolizumab in human serum were measured using ELISA, with the lower limit of quantification of 60 ng/mL.

The PK analysis included the maximum serum atezolizumab concentration, the area under the serum concentration–time curve from time zero to infinity, clearance (CL), terminal elimination half-life (t_1/2_), volume of distribution at steady state, and CV. These data were analyzed by the linear log trapezoidal method with Phoenix WinNonlin version 6.2 (Pharsight Corporation, Mountain View, CA, USA).

### PD-L1 expression level

PD-L1 expression was evaluated by the VENTANA SP142 antibody (Ventana Medical Systems, Inc., Tucson, AZ). TC scores were assigned based on the percentage of PD-L1-expressing TC (TC3: ≥50 %; TC2: ≥5 % and <50 %; TC1: ≥1 % and <5 %; TC0: <1 %). IC scores were assigned based on the percentage of PD-L1-expressing IC within the tumor area (IC3: ≥10 %; IC2: ≥5 % and <10 %; IC1: ≥1 % and <5 %; IC0: <1 %).

### Statistical analysis

The planned sample size was six to 12 patients, selected to allow evaluation of the safety profile and tumor response and to investigate PK and PD-L1 expression.

## Results

### Patients

Six patients were enrolled between September 2013 and October 2013. The patients’ characteristics are listed in Table 1. Three patients were men and three were women, and their median age was 51 years (range, 41–69). Three patients had non-small cell lung cancer (NSCLC; adenocarcinoma), and one patient each had pancreatic cancer, melanoma, or thymic cancer. The ECOG PS was 0 in two patients and 1 in four patients. No patients had TC3 or IC3 tumors. The patient with thymic carcinoma had IC2 and TC2 scores, the patient with pancreatic cancer had IC1 and TC0 scores, and the patient with melanoma had IC0 and TC0 scores. One of three patients with NSCLC had IC1 and TC0 scores, another had IC0 and TC0 scores, and the remaining patient’s NSCLC was not assessable for IC or TC.

**Table 1:** Patient characteristics

Characteristic	No. of patients
Total no. of patients	6
Age in years, median (range)	51 (41–69)
Sex	
Male/female	3/3
ECOG-PS	
0/1	2/4
No. of prior chemotherapies	
1/2/≥3	1/1/4
Primary tumor	
NSCLC	3
Melanoma	1
Pancreatic cancer	1
Thymic cancer	1
PD-L1 status	
IC score	
2/1/0/NA	1/2/2/1
TC score	
2/1/0/NA	1/0/4/1

*IC* tumor-infiltrating immune cells, *NA* not assessable, *NSCLC* non-small cell lung cancer, *PD*-*L1* programmed death-ligand 1, *TC* tumor cells

Three patients were enrolled in Cohort 1, and three were enrolled in Cohort 2. The median duration of treatment was 53.9 weeks (range, 13.1–61.1) in Cohort 1 and 56.1 weeks (12.1–56.9) in Cohort 2. Half of all patients were treated for more than 1 year, and the patient with thymic cancer remained on treatment as of December 2015 (more than 2 years of treatment). The median relative dose intensity (actual dose/planned dose × 100) was 94.7 % (range, 91.3 %–98.1 %) in Cohort 1 and 98.80 % (96.2 %–100.3 %) in Cohort 2.

Atezolizumab was started in all six enrolled patients, and three of the six patients (one in Cohort 1 and two in Cohort 2) were still participating in the study at the time of data cutoff. Three patients withdrew due to inadequate responses.

### Safety and tolerability

All six patients experienced AEs (24 individual events in Cohort 1 and 18 in Cohort 2). One event (influenza-like illness) was reported as serious. No events resulted in death or discontinuation of study treatment, although three patients experienced AEs that led to suspension of study treatment (influenza-like illness and increased ALP in one patient each in Cohort 1 and pneumonia in one patient in Cohort 2; influenza-like illness and increased ALP were considered to be related to atezolizumab). All events were grade 1 or 2. Overall, rash was the most common related AE, followed by increased aspartate aminotransferase, alanine aminotransferase, and ALP and headache (Table 2). No DLTs were reported; therefore, the maximum tolerated dose was not reached. Overall, no new safety signals were observed.

**Table 2:** AEs in two or more patients

Atezolizumab dose	10 mg/kg		20 mg/kg		Total	
No. of patients	*n* = 3		*n* = 3		*n* = 6	
AE grade^a^	1	2	1	2	1	2
Rash	2	0	1	0	3	0
AST increased	1	0	1	0	2	0
ALT increased	1	0	1	0	2	0
ALP increased	0	1	1	0	1	1
Headache	2	0	0	0	2	0

*AE* adverse event, *ALP* alkaline phosphatase, *ALT* alanine aminotransferase, *AST* aspartate aminotransferase

^a^All AEs were grade 1 or 2

### Pharmacokinetics

**Fig. 1:**
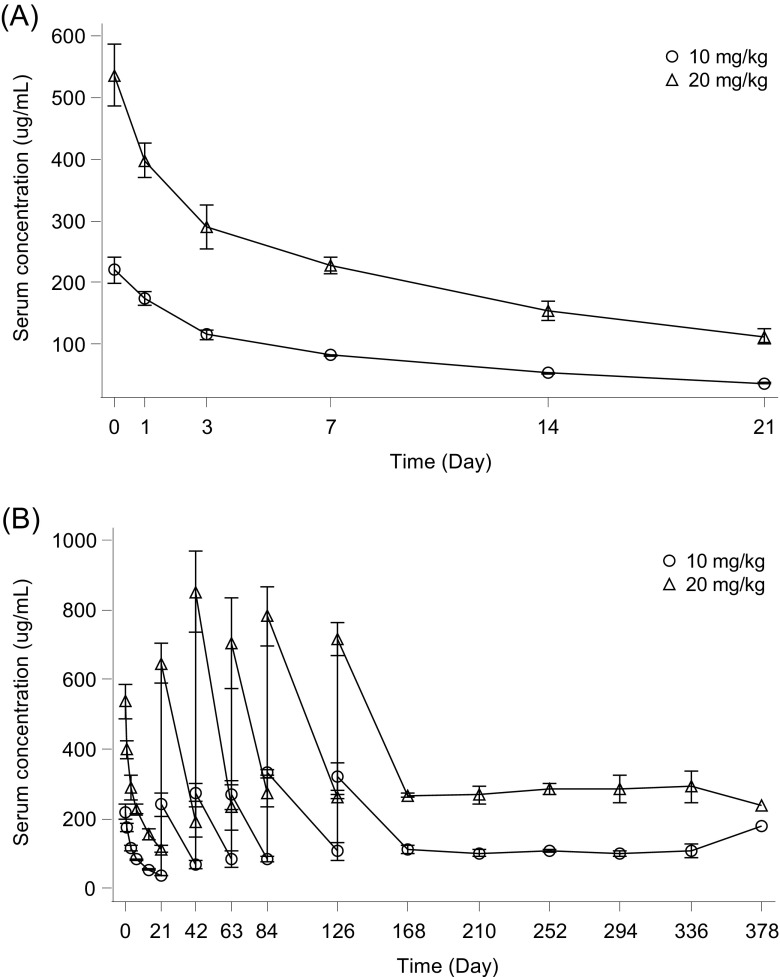
Mean ± SD serum atezolizumab concentration–time profile (semi-log scale) at first infusion (**a**) and over 18 cycles (**b**)

The PK evaluation was performed in all six patients. The PK profile of atezolizumab is summarized in Table 3, and the mean serum concentration–time profiles of atezolizumab are illustrated in Fig. 1. Mean ± SD trough levels of atezolizumab during Cycle 1 in Cohorts 1 and 2 were 36.8 ± 1.35 and 113 ± 11.3 μg/mL, respectively. The trough values from Cycle 3 to Cycle 18 were 84.6 to 179 μg/mL and 232 to 292 μg/mL, respectively. The doses for each patient were 446, 469, and 713 mg in Cohort 1 and 1088, 1446, and 1626 mg in Cohort 2. CL and t_1/2_ were similar in the two cohorts. The mean ± SD CL was 236 ± 57.2 mL/day in Cohort 1 and 213 ± 60.9 mL/day in Cohort 2, and the t_1/2_ values were 11.7 ± 0.969 and 13.0 ± 1.32 days, respectively.

**Table 3:** Pharmacokinetic parameters of atezolizumab

Parameter	Unit	*n*	Mean	SD	CV (%)	Geometric mean	Geometric CV (%)
Cohort 1: 10 mg/kg							
C_max_	μg/mL	3	220	21.9	9.95	219	10.3
AUC_inf_	day•μg/mL	3	2290	101	4.43	2290	4.49
CL	mL/day	3	236	57.2	24.2	232	23.3
t_1/2_	day	3	11.7	0.969	8.31	11.6	8.51
V_ss_	mL	3	3720	1140	30.6	3620	29.3
Cohort 2: 20 mg/kg							
C_max_	μg/mL	3	536	49.4	9.22	534	9.14
AUC_inf_	day•μg/mL	3	6630	668	10.1	6610	10.4
CL	mL/day	3	213	60.9	28.6	207	29.3
t_1/2_	day	3	13.0	1.32	10.1	13.0	10.2
V_ss_	mL	3	3820	718	18.8	3780	18.8

*AUC*_*inf*_ area under the serum concentration–time curve from time zero to infinity, *CL* clearance, *C*_*max*_ maximum serum concentration, *t*_*1*/*2*_ elimination half-life, *V*_*ss*_ volume of distribution at steady state

### Tumor response

**Fig. 2:**
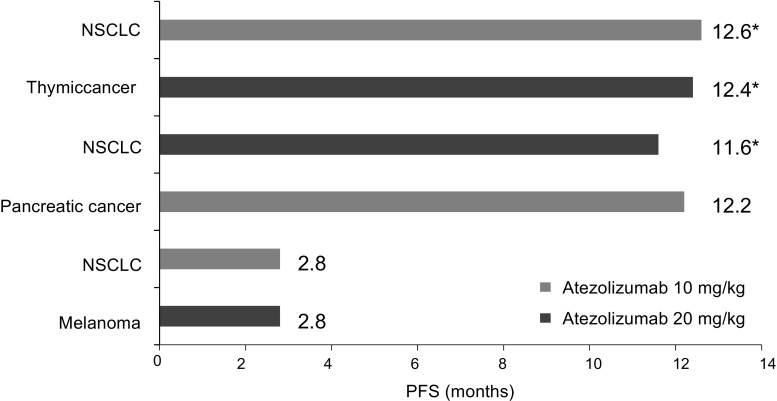
Duration of progression-free survival * Censored observation. *NSCLC* non-small cell lung cancer, *PFS* progression-free survival

All six patients were evaluable for anti-tumor response with RECIST version 1.1. The best overall response was stable disease in all six patients (Table 4). Progression-free survival was >12 months in three of six patients who remained on treatment at data cutoff (Fig. 2).

**Table 4:** Objective tumor response and duration of treatment

Dose level	No. of cycles	Primary tumor	PD-L1 expression		Best overall response
(mg/kg)			IC score	TC score	
10	20	NSCLC	NA	NA	SD
20	19	Thymic cancer	2	2	SD
20	18	NSCLC	0	0	SD
10	17	Pancreatic cancer	1	0	SD
10	4	NSCLC	1	0	SD
20	4	Melanoma	0	0	SD

*IC* tumor-infiltrating immune cells, *NSCLC* non-small cell lung cancer, *PD*-*L1* programmed death-ligand 1, *TC* tumor cells, *NA* not assessable

## Discussion

Atezolizumab is an engineered immunoglobulin 1 (IgG1) monoclonal antibody that targets the PD-L1/PD-1 and PD-L1/B7.1 (CD80) pathways. We conducted a phase I dose-finding study of atezolizumab in Japanese patients with solid tumors. Atezolizumab at 10 and 20 mg/kg every 3 weeks was well tolerated in this Japanese patient population, since no AEs met the definition of DLT. The safety profile was consistent with that seen in a previous non-Japanese phase Ia clinical study of atezolizumab [9].

The frequency of AEs is only a guide, because this was a small study. Overall, the AE profile of atezolizumab in this study was similar to that observed previously in the larger phase Ia study in Western patients [9], and no new safety concerns were identified. No AEs required medical treatment. The most frequently reported AE was fatigue in previous non-Japanese clinical studies of atezolizumab and of the anti-PD-1 antibodies nivolumab and pembrolizumab [9, 17–19]; however, no fatigue was observed in this study. The accumulation of AE data in Japanese patients might be important in the future.

As with safety, serum atezolizumab concentrations in Cycle 1 were similar to those seen in the previous non-Japanese phase Ia clinical study of atezolizumab [9]. In contrast, the mean t_1/2_ was shorter in our study (<2 weeks vs ≈ 3 weeks). In our study, the serum atezolizumab concentration (mean ± SD) was 36.8 ± 1.35 μg/mL or higher when atezolizumab was administered at a dose of 10 mg/kg every 3 weeks. The doses in each patient in the 10 mg/kg dose group were 446, 469, and 713 mg, respectively, which were lower than the 1200 mg dose being investigated in phase III clinical studies of atezolizumab in solid tumors with study sites in Japan (ClinicalTrials.gov; NCT02302807, NCT02420821, NCT02367794, NCT02366143, and NCT02409342). Therefore, the serum atezolizumab concentration would also be 36.8 ± 1.35 μg/mL or higher if the dose in each patient of our study was 1200 mg. The 1200 mg dose is expected to maintain a higher concentration than 10 mg/kg, which was a sufficient dose in the phase Ia study [9] and is lower than the 20 mg/kg dose in our study, which showed no new safety signals. CL, determined from the serum atezolizumab concentration at the first infusion using a noncompartmental analysis, was similar in Cohorts 1 and 2. CL did not tend to increase or decrease in a specific direction when the dose was changed and was in the range of 156 to 302 mL/day. Therefore, CL does not change in the dose range studied here (446–1626 mg; 10 or 20 mg/kg). Although our study was limited in terms of the number of patients (*N* = 6), systemic atezolizumab exposure is known to be unaffected by differences in region or race [20].

The best overall response was stable disease in all six patients, and progression-free survival was >12 months in three patients. The response rate and duration of response with atezolizumab were longer than those in other Japanese phase I studies for patients after standard therapy [21]. In a previous global phase Ia study, there appeared to be an association between the anti-tumor response with atezolizumab and the expression of PD-L1 in pretreatment samples. The association of response to atezolizumab treatment and IC PD-L1 expression was statistically significant, whereas the association with TC PD-L1 expression was not statistically significant across all tumor types in the phase Ia study [9]. However, in our study, the level of PD-L1 expression and anti-tumor response did not follow any specific pattern due to the small number of patients. Evidence of anti-tumor response was limited in this study; therefore, a relationship between the level of PD-L1 expression and anti-tumor response could not be determined in Japanese patients. The other exploratory objectives were immunogenicity, T/B/NK cell counts, and Fcγ receptor III polymorphism. We expected to detect the utility of T/B/NK cell counts as a biomarker and the relationship between AEs and Fcγ receptor III polymorphism. However, no specific data were available in this small study (data not shown). Additional research is needed to identify adequate patients using reliable biomarker to predict patient benefits.

In conclusion, the results of this study confirm that atezolizumab is well tolerated in Japanese patients at a dosage up to 20 mg/kg q3w. At this dose level, atezolizumab has an acceptable toxicity profile with a PK profile similar to that seen in a US phase I study. Overall, results obtained in this study support the participation of Japanese patients in ongoing pivotal global studies of atezolizumab in advanced solid tumors (ClinicalTrials.gov; NCT02302807, NCT02420821, NCT02367794, NCT02366143, and NCT02409342).
